# Change in the Stations of Buffalo Physicians in the War

**Published:** 1861-11

**Authors:** 


### CHANGE IN THE STATIONS OF BUFFALO PHYSICIANS IN THE WAR.

We think that our home subscribers will be pleased to keep posted in the fortunes of our Buffalo Surgeons in the government service. Since our last the following changes have been made in their stations :

Dr. N. L. BATES has been transferred from the Naval Hospital, Brooklyn, to the New Gun Boat Seneca, 8 guns, and sailed with the great expedition for —— Sesesh.

Acting Assistant Surgeon G. J. SWEET, has been ordered to the purchased steamer "Isaac Smith," and has sailed with the squadron.

Dr. FLAGG returned on a visit, from a cruise in the Gulf, but was suddenly ordered off on his ship, in pursuit of the fugitive ambassadors from Jeff's Kingdom. The Dr. saw Dr. Whitehead at Key West, on a store ship. He was looking well and had *gained flesh.*

Dr. Howell was at Pass L'Outre, (on the "Richmond,") he had had a severe attack of pulmonary hæmorrhage, but was recovering. It also affords us pleasure to record the names of two other graduates of the Buffalo Medical College, Dr. F. J. Bancroft, who has successfully passed the Army Medical Board, and is Assistant Surgeon in that branch of the service, and Dr. Charles S. Boynton, Assistant Surgeon U. S. Gun Boat service, on the Western Rivers. B.

